# Fetal Appendiceal Perforation Masquerading as Meconium Peritonitis: A Report of a Rare Case

**DOI:** 10.7759/cureus.60576

**Published:** 2024-05-18

**Authors:** Nimisha Singh, Ayush K Singh

**Affiliations:** 1 General Surgery, Himalayan Institute of Medical Sciences, Dehradun, IND; 2 Plastic and Reconstructive Surgery, Jawaharlal Nehru Medical College (JNMC) Aligarh Muslim University (AMU), Aligarh, IND

**Keywords:** dichorionic-diamniotic twins, antenatal, exploratory laparotomy, meconium peritonitis, fetal appendiceal perforation

## Abstract

Intrauterine appendicular perforation leading to meconium peritonitis is exceptionally rare, with few reported cases in the literature. This case underscores the diagnostic challenges and high mortality associated with neonatal appendicular perforation. Neonatal appendicitis and subsequent perforation are uncommon due to the funnel shape of the fetal appendix, which reduces susceptibility to luminal obstruction. While advances in neonatal care and diagnostic modalities have improved outcomes, challenges persist in timely diagnosis and management. We present the case of a preterm infant, one of dichorionic-diamniotic (DCDA) twins delivered via cesarean section, who developed gross abdominal distension and respiratory distress shortly after birth. Diagnostic abdominocentesis revealed meconium-stained fluid, prompting further investigation with imaging and subsequent exploratory laparotomy. Extensive adhesions and cecal perforation were observed, necessitating a cecostomy. Despite interventions, the infant's condition deteriorated, leading to a fatal outcome. Intrauterine appendicular perforation leading to meconium peritonitis is a rare and difficult-to-diagnose condition. Antenatal suspicion and early surgical intervention are crucial for improving outcomes. Factors contributing to neonatal appendicular perforation include ischemia, obstruction, and infective etiologies. Neonatal appendicular perforation is a rare but life-threatening condition requiring a high index of suspicion for prompt diagnosis and management. Advances in diagnostic tools and antenatal monitoring have contributed to improved outcomes, highlighting the importance of considering this diagnosis in cases of unexplained neonatal abdominal distension.

## Introduction

Over a century has passed since Wangenstein [1] first hypothesized and demonstrated that appendicitis is caused by luminal obstruction. Appendicitis leading to appendicular perforation in the neonatal period is exceedingly rare, with fewer than 100 cases reported in the literature. The rarity of appendicular perforation and its consequences can largely be attributed to the funnel shape [1] of the fetal appendix, which is believed to be resilient to obstruction and blockage.

The recumbent position of neonates and their primary diet of milk, which leaves behind no undigested residue, contribute to the low susceptibility of neonates to appendicitis and subsequent perforation.

Neonatal appendicitis tends to occur more frequently in preterm infants, with an increased risk of perforation and rapid progression to meconium peritonitis. The subtle signs and low index of suspicion often lead to delays in diagnosis and management. The mortality rate of neonatal appendicitis was as high as 78% between 1901 and 1975 [2], which subsequently declined to 33% during 1976-1984 due to rapid advances in antibiotic therapy, neonatal intensive care, and diagnostic modalities. The further decrease in the death rate to 28% during 1985-2003 represented only modest improvement [2].

Given its rarity and challenges in diagnosis, neonatal appendicitis is frequently overlooked during pregnancy. Here, we present a case report of appendicular perforation.

## Case presentation

A preterm infant, aged 34 weeks and five days and the elder of dichorionic-diamniotic (DCDA) twins, was delivered by a 22-year-old female via cesarean section in a hospital in Dehradun, India. On the same day after delivery, the infant presented with complaints of gross abdominal distension and respiratory distress. Subsequently, the patient was intubated due to severe respiratory distress.

**Figure 1:**
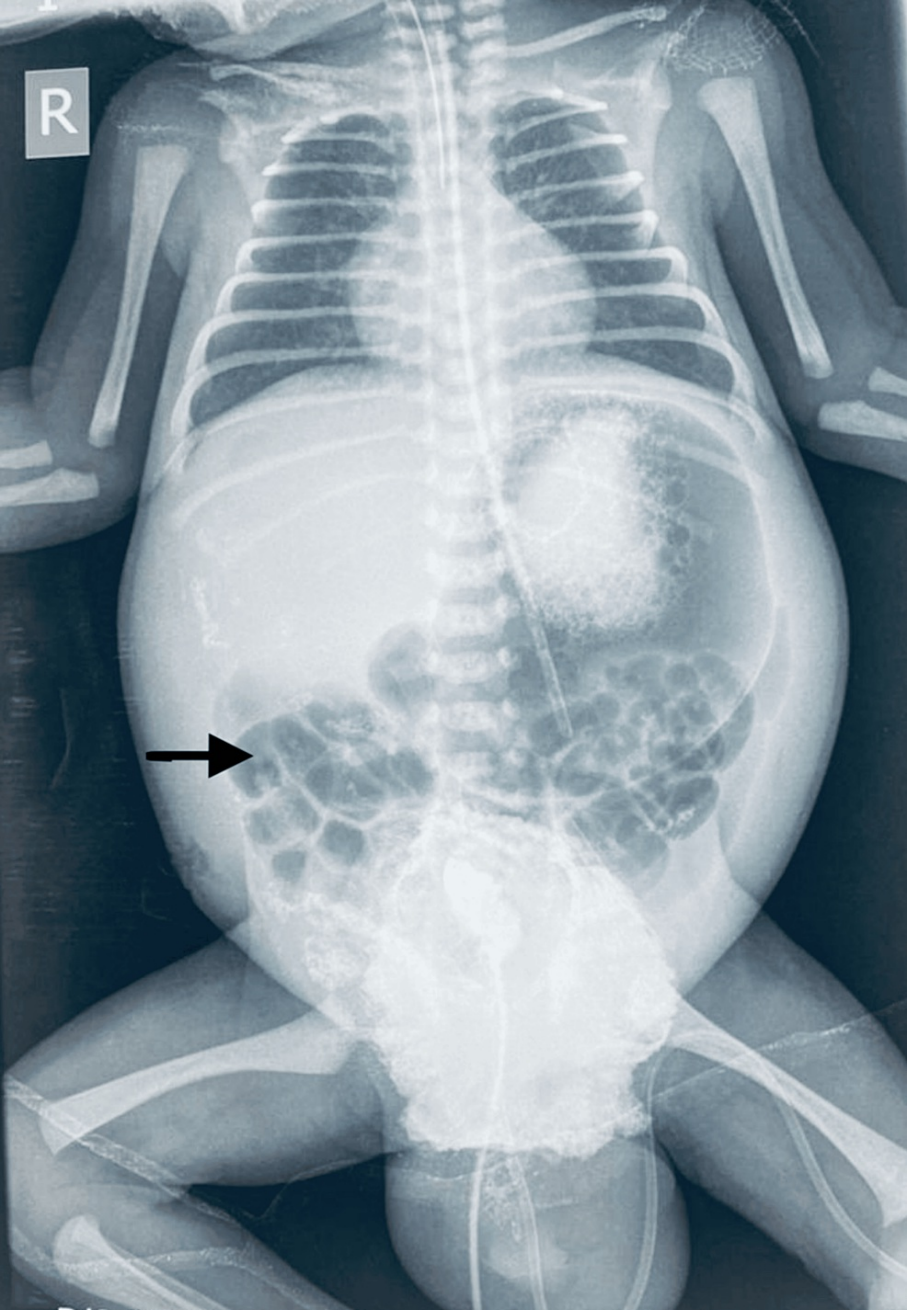
Gross dilation of bowel loops (marked with black arrow) suggestive of abdominal distention

Upon physical examination, the infant exhibited abdominal distention, along with tenderness and a distended scrotum, resembling a hydrocele. However, no occult blood was found in the stool. Sepsis screening yielded gram-positive cocci for which he was started on ampicillin and vancomycin. The baby was on nasogastric suction and started on total parenteral nutrition (TPN). Abdominal X-ray imaging revealed dilation of the bowel loops leading to abdominal distension (Figure 1).

On the second day, bedside ultrasonography revealed gross ascites with calcifications in the mesentery and omentum, suggestive of pneumoperitoneum. Diagnostic and therapeutic abdominocentesis confirmed meconium-stained fluid, indicating meconium peritonitis and providing symptomatic relief. 

**Figure 2:**
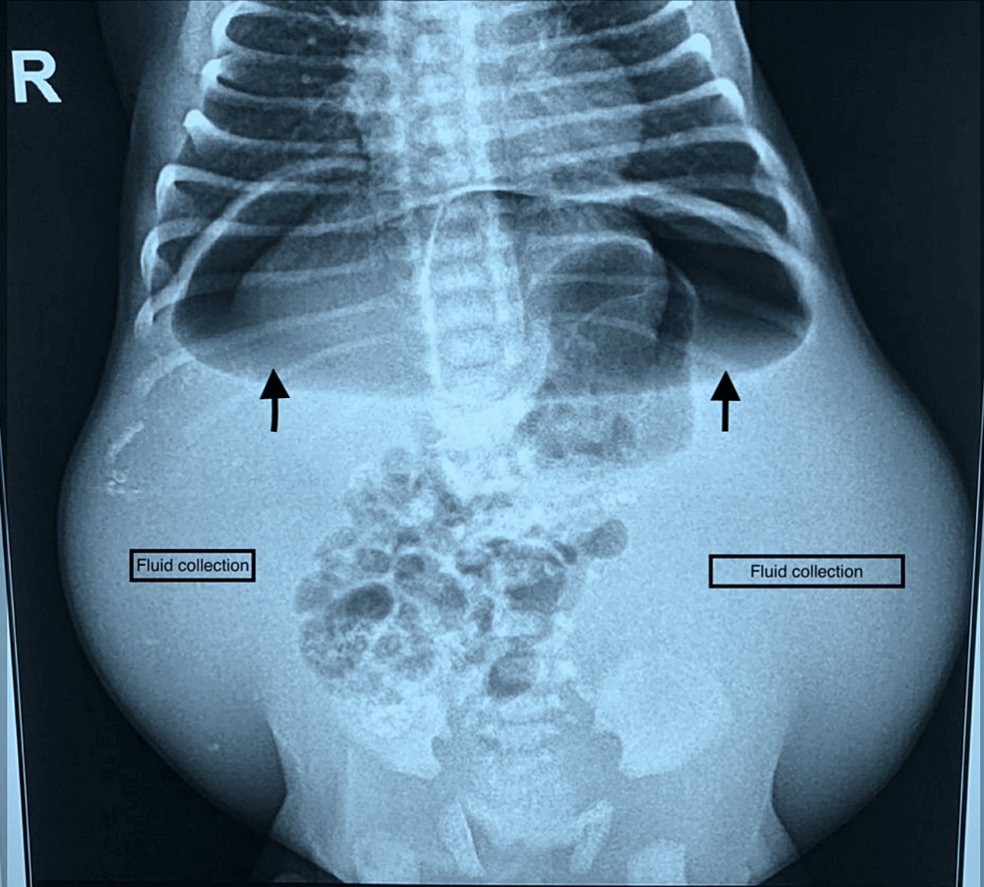
Abdominal X-ray imaging revealed air-fluid levels, gas under the diaphragm (marked with black arrow), and gross fluid accumulation in the abdomen and scrotum

On day 3, abdominal X-ray imaging revealed air-fluid levels, gas under the diaphragm, and gross fluid accumulation in the abdomen and scrotum (Figure 2). The neonate was initially managed conservatively considering their overall condition. However, suspicions of a sealed perforation arose as the neonate's condition gradually improved. A subsequent water-soluble contrast enema identified extravasation of dye in the abdominal cavity and scrotum confirming perforation peritonitis, prompting immediate exploratory laparotomy.

During exploratory laparotomy, extensive adhesions, cecal perforation, and edematous bowel loops were observed. The appendix was not visualized, and a perforated cecal segment necessitated a cecostomy. The patient's general condition improved over time, and by postoperative day 2, the neonate was successfully extubated. However, he subsequently developed sclerema, prompting antibiotic adjustment based on culture sensitivity findings. Despite receiving platelets and packed red blood cell transfusions for severely low platelet counts (10,000/cu mm) and hemoglobin levels (11 gm/dl) (Table 1), the neonate experienced recurrent severe respiratory distress and endotracheal bleeding. This deterioration ultimately led to bradycardia, requiring cardiopulmonary resuscitation (CPR) according to Advanced Trauma Life Support (ATLS) guidelines. Regrettably, despite resuscitative efforts, the neonate passed away on postoperative day 3 due to the severity of the illness.

**Table 1:** Relevant deranged blood profile

Parameter	Patient's value	Normal range (neonates)
Hemoglobin	11 g/dl	14-24 g/dl
Total leucocyte count	32,000/cu mm	9,000-30,0000/cu mm
Platelet	10,000/cu mm	150,000-450,000/cu mm
International normalized ratio	1.7	0.9-1.1

## Discussion

Intrauterine perforation of the appendix leading to meconium peritonitis is an extremely rare occurrence, with only a few cases reported in the medical literature. The rarity of appendicular infection and perforation can be attributed to its shape, resembling a funnel with a wide open base, which prevents blockage. To the best of our knowledge, this case report represents the first documented instance in English literature where one of the twins in a DCDA pregnancy had fetal perforation of the appendix resulting in meconium peritonitis. Shaul suggested that the mortality rate of neonatal appendicitis ranges from 50% to 88% [3]. It has male predilection in approximately 75% of cases, and 25-50% of cases involve premature infants [4]. A high index of suspicion and clinicians' knowledge of this condition may aid in antenatal suspicion and early intervention, potentially saving lives. The fetal pathophysiology of perforated appendicitis differs from that in adults and may occur secondary to ischemia, obstruction (such as colonic or anal atresia or Hirschsprung's disease), mid-gut volvulus [5,6], infective diseases (like chorioamnionitis or cytomegalovirus), primary vascular insufficiency, or congenital aplasia of the muscularis mucosa or enteric duplication.

The male baby, first of the twin pair, was born with a distended abdomen, which caused respiratory distress and possible scrotal swelling. An abdominal X-ray revealed significant ascites with the possibility of gas under the diaphragm, indicative of bowel perforation. Ascitic fluid drainage confirmed the presence of meconium ascites and temporarily relieved the child's respiratory distress. During exploratory laparotomy, marked bowel loop adhesions were observed, particularly in the cecal region. A perforation at the base of the cecum was identified, and the appendix was not visualized, necessitating a cecostomy at that site.

Various acute abdominal symptoms, including abdominal distention, vomiting, and fever, may be present; however, preoperative diagnosis remains challenging. Wang et al. reported two similar cases where antenatal monitoring of dimensions and observations of distension and ileus led to suspicion of meconium peritonitis [1]. Antenatal diagnosis of meconium peritonitis is now possible with improved imaging techniques such as prenatal graded compression ultrasound, which is highly accurate, aiding in early diagnosis and providing crucial information; however, its sensitivity varies and relies heavily on the operator's skill [7]. Abdominal X-rays in various positions (supine, upright, and lateral) are usually insufficient for identifying intraperitoneal free gas. Routine CT scans are not advised for neonates due to the potential harm from unnecessary radiation exposure. Surgery is indicated when signs of peritonitis, positive abdominocentesis, or severe imaging and laboratory findings are present. Early exploratory laparotomy is essential. Extensive adhesions and calcifications may be encountered. If bowel repair is not feasible, a cecostomy with drainage may be necessary, and in severe cases, an ileocecal resection may be required. Given the high mortality rate associated with this condition, early diagnosis, ideally prenatal, and prompt surgical intervention are imperative for improving outcomes.

## Conclusions

Appendicular perforation should be suspected in all cases of intrauterine or neonatal unexplained abdominal distension with associated symptoms. Graded compression ultrasound plays an important role in aiding early diagnosis and providing crucial information for the treatment of perinatal infants. Early suspicion and recognition of this condition are crucial for optimizing outcomes and reducing mortality rates associated with newborn abdominal emergencies.

## References

[1] Wang Y, Wu Y, Guan W, Yan W, Li Y, Fang J, Wang J (2018). Meconium peritonitis due to fetal appendiceal perforation: two case reports and a brief review of the literature. BMC Pediatr.

[2] Karaman A, Cavuşoğlu YH, Karaman I, Cakmak O (2003). Seven cases of neonatal appendicitis with a review of the English language literature of the last century. Pediatr Surg Int.

[3] Shaul WL (1981). Clues to the early diagnosis of neonatal appendicitis. J Pediatr.

[4] Ruff ME, Southgate WM, Wood BP (1991). Radiological case of the month. Am J Dis Child.

[5] Martin LW, Perrin EV (1967). Neonatal perforation of the appendix in association with Hirschsprung's disease. Ann Surg.

[6] Saeki I, Yamanouchi T, Tanaka S, Kawanami T, Mori R, Zaizen Y (2012). Neonatal appendicitis mimicking intestinal duplication: a case report. J Med Case Rep.

[7] Fu F, Song X, Huang F, Yuan H, Xiao L (2022). Fetal meconium peritonitis: a clinical study of nine cases. Comput Intell Neurosci.

